# Is lethality different between males and females? Clinical and gender differences in inpatients suicide attempters

**DOI:** 10.1192/j.eurpsy.2023.700

**Published:** 2023-07-19

**Authors:** I. Berardelli, E. Rogante, S. Sarubbi, D. Erbuto, M. Cifrodelli, C. Concolato, M. Pasquini, D. Lester, M. Innamorati, M. Pompili

**Affiliations:** ^1^Sapienza University of Rome, Rome, Italy; ^2^Stockton University School, Galloway, United States; ^3^European University of Rome, Rome, Italy

## Abstract

**Introduction:**

According to the gender paradox,in suicidology an important sex difference has been reported with a preponderance of females in nonfatal suicidal behavior and a preponderance of males in completed suicide.The lethality of suicidal behavior in females is lower most likely because males choose more violent suicide methods.Furthermore,women more frequently present traditional risk factors for suicide than do men,including depression,childhood sexual abuse, and prior suicidal ideation and attempts.

**Objectives:**

The purpose of this study was to explore possible clinical differences between male and female psychiatric inpatients who had recently attempted suicide.We hypothesized that clinical characteristics such as psychiatric diagnosis,the methods and lethality of the suicide attempt,the history of suicide attempts,age at onset of psychiatric illness,the presence of substance or alcohol use and the length of stay differ between male and female suicide attempters.

**Methods:**

The study included 177 adult inpatients at the University Psychiatric Clinic, Sant’Andrea Hospital, Sapienza University of Rome hospitalized following a suicide attempt, between January 2018 and May 2022.Clinical features assessed included psychiatric diagnosis, method and lethality of suicide attempts using the Risk-Rescue Rating Scale, the history of suicide attempts, age at onset of psychiatric illness, the presence of substance or alcohol use, and the length of stay.All statistical analyses were performed with the Statistical Package for Social Sciences(SPSS 27.0).

**Results:**

Males and females differed according to the method used for suicide attempt(x23=10.96,p<0.05),the scores for risk and rescue(t175=2.55,p<0.05;t146.6=-1.99,p<0.05,respectively),and the length of stay(U= 3084.5,p<0.05).Females were more likely to use drug/poisoning ingestion as method for suicide attempt than were males(72.8% vs.51.4%),whereas males were more likely to use hanging than were females(20.3% vs. 6.8%).The risk score was higher for males(3.76±0.68)than for females(3.49±0.72),and the rescue score was higher for females than for males(2.79±1.09 vs. 2.43±1.22).Finally,the length of stay was longer for males than for females(10.66±8.09 vs. 8.25±6.48).These results confirm the role of difference in suicide methods used by males and females for explaining the “gender paradox.”

**Conclusions:**

The present study illustrates the usefulness of the Risk-Rescue Rating Scale which is a descriptive and quantitative method of assessing the lethality of suicide attempts.Identifying sex related characteristics of suicide risk in patients is important for implementing specific suicide prevention strategies to reduce suicidal intent, psychological pain and rehospitalization in patients with psychiatric disorders.Men and women may need different strategies for the prevention of future suicidal behavior.

**Disclosure of Interest:**

None Declared

